# A Chromosome-Level Genome Assembly of the Pygmy Mole Cricket *Xya riparia*

**DOI:** 10.1093/gbe/evac001

**Published:** 2022-01-06

**Authors:** Xiaolei Feng, Nan Yang, Qilu Wang, Hao Yuan, Xuejuan Li, Muhammad Majid, Xue Zhang, Chengquan Cao, Yuan Huang

**Affiliations:** 1 School of Life Sciences, Shaanxi Normal University, Xi’an, Shaanxi, China; 2 School of Basic Medical Sciences, Xi’an Medical University, Shaanxi, China; 3 College of Life Sciences, Leshan Normal University, Sichuan, China

**Keywords:** Orthoptera, Tridactyloidea, reference genome, gene annotation, nanopore sequencing, Hi-C

## Abstract

The pygmy mole cricket *Xya riparia* (Orthoptera: Tridactyloidea) is rarely studied or widely known. Some species of pygmy mole crickets, however, not only have a potential ecological value but are also important in the study of the evolution of the orthopteran genome and its phylogenetic relationships. The genome resources of pygmy crickets are limited and there are currently no publications referencing this species’ genome. In this study, we assembled a reference genome of *X. riparia* at the chromosomal level using nanopore sequencing and Hi-C technology. An *X. riparia* genome of 1.67 Gb was successfully assembled from 164.01 Gb of nanopore sequencing data. The genome assembly showed a completeness of 98.97% benchmarking universal single-copy orthologs with a contig N50 of 4.18 Mb and the longest contig being 18.84 Mb. The contigs were clustered, ordered, and correctly oriented on six pseuchromosomes, which covered 95.63% of the genome assembly through Hi-C data with a scaffold N50 of 319.1 Mb and the longest scaffold being 397.8 Mb. Repeat sequences accounted for 42.88% of the whole-genome assembly. A total of 60,847 noncoding RNAs were detected. Moreover, 16,468 (87.91%) of the genes were functionally annotated. As this is the first high-quality reference genome of *X. riparia* at the chromosomal level, it will undoubtedly serve as a valuable resource for ecological, biological, and genetic research on pygmy mole crickets as well as for general research on Orthoptera’s genome evolution and phylogenetic relationships.

Significance The high-quality whole-genome assembly of the pygmy mole cricket *Xya riparia* at the chromosomal level is the first reference genome of Tridaetyloidea, order Orthoptera. It is an important resource to understand the evolution of the genome’s size and the phylogenetic relationships of orthopteran insects.

## Introduction

Pygmy mole crickets (Orthoptera: Caelifera: Tridactyloidea) evolved from a group of ancient species, which can be traced back to the Cretaceous period (Cao et al. 2019). They are widely distributed in the world and can be found almost anywhere except the poles (http://orthoptera.speciesfile.org/Common/basic/Taxa.aspx?TaxonNameID=1100051, last accessed September 2021). The body length of pygmy mole crickets is 3.8–10 mm (Woo 2021), which is much smaller than other orthopteran species in the suborder Caelifera. Compared with other caeliferan species that live in fields and feed on crops (Bullen 1966), pygmy mole crickets often live on riverbanks (Song 2018) and mainly feed on moss (Kuravova and Kocarek 2016; Ugolini 2021). The diet of pygmy mole crickets makes them less agriculturally harmful than their caeliferan counterparts, and thus they have not been the focus of much research aimed at minimizing the ecological impact of orthopteran species. Some of the pygmy mole cricket’s biological characteristics, however, may have potential bionic values in engineering. Previous studies on pygmy mole crickets revealed that they can jump both far and high to avoid predators like tiger beetles and can also jump from the water’s surface to avoid fish predators (Burrows and Picker 2010). Their jumping mechanism and their paddles can be mimicked in order to propel bionic subaquatic robotic vehicles (Siddall and Kovač 2014; Sudo et al. 2015; Mo et al. 2020). Moreover, previous studies have also revealed that pygmy mole crickets are quite sensitive to floodplain regulation, and some species have already become extinct because of changes in the river systems (Münsch et al. 2013). This species’ sensitivity, therefore, has potential ecological value in monitoring the changes of dynamic river systems.

Orthoptera is the order with the largest genome within the class Insecta, and this order’s genome size varies from 1.52 to 18.23 Gb (Lai and Sun 2003). The reasons behind Orthoptera’s large genome remain unclear. Presently, the available genome resources of orthopteran insects is limited and only a few studies on the genome of orthopteran insects have been conducted (Wang et al. 2014; Blankers et al. 2018; Verlinden et al. 2020). Further research to obtain high-quality genome data is the only way to determine the cause of the orthopteran order’s genome enlargement and to establish more robust phylogenetic relationships among species contained in the order Orthoptera.

In this study, we collected living females of *X. riparia* (supplementary fig. 1, Supplementary Material online) in Leshan, Sichuan Province, China and assembled the genome at the chromosomal level using Illumina sequencing, nanopore sequencing, and Hi-C technology. This is the first reference genome of Tridactyloidea with a high-quality genome assembly, detailed descriptions, and gene annotations. This reference genome is valuable for future studies involving comparative genomic analysis, population genomics, and phylogenetic evolution.

## Results and Discussion

### Genome Size Estimation

In order to estimate the genome size of *X. riparia*, a total of 113.02 Gb of Illumina sequencing data with a coverage of about 65× was used for *k*-mer (*k* = 21 in this case) analysis. A total of 97,899,858,172 *k*-mers were obtained. By discarding the abnormal *k*-mers, 90,170,405,160 *k*-mers were then used to estimate the genome’s size. According to the plot, the highest peak of the *k*-mers was detected at a *k*-mer depth of 52 (supplementary fig. 2, Supplementary Material online). The *k*-mer analysis showed that the genome size of *X. riparia* was estimated to be 1.71 Gb. The heterozygosity and GC content were 1.32% and 34.94%, respectively, which indicated that the genome of *X. riparia* was high in both heterozygosity and complexity.

### Nanopore Sequencing and Assembly

A total of 164.01 Gb of clean data was obtained after nanopore sequencing. The sequencing depth was about 98×. After filtering out the low-quality reads, as many as 5,933,413 reads were obtained, with a mean length of 27,641 bp and an N50 length of 38,027 bp, respectively. After error correction and assembly, the final length of the *X. riparia* genome was 1.67 Gb with a contig N50 of 4.33 Mb and the longest contig being 19.91 Mb, which was close to the estimated size of the final length obtained from the *k*-mer analysis (1.71 Gb).

We compared the genome assembly with benchmarking universal single-copy orthologs (BUSCO) in order to assess its completeness. A total of 1,055 (98.97%) complete BUSCOs were identified, including 1,011 (94.84%) single BUSCOs, 44 (4.13%) duplicated BUSCOs, three (0.28%) fragmented BUSCOs, and eight (0.75%) missing BUSCOs (table 1). The results of the BUSCO comparison showed a high degree of completeness in the genome assembly.

**Table 1:** Summary of *Xya riparia* Genome Assembly and Completeness Assessment

Genome assembly	Estimated genome size	1.71 Gb
	Assembly size (scaffold)	1.66 Gb
	Assembly size (contig)	1.67 Gb
	Hi-C anchored rate	95.63%
	Contig number	1,030
	Contig N50	4.18 Mb
	Longest contig	18.8 Mb
	Scaffold number	467
	Scaffold N50	319.1 Mb
	Longest scaffold	397.8 Mb
	GC content	34.84%
BUSCO	Complete BUSCOs (C)	1,055 (98.97%)
	Complete and single-copy BUSCOs (S)	1,011 (94.84%)
	Complete and duplicated BUSCOs (D)	44 (4.13%)
	Fragmented BUSCOs (F)	3 (0.28%)
	Missing BUSCOs (M)	8 (0.75%)

### Chromosome-Level Genome Assembly

**Fig. 1.**
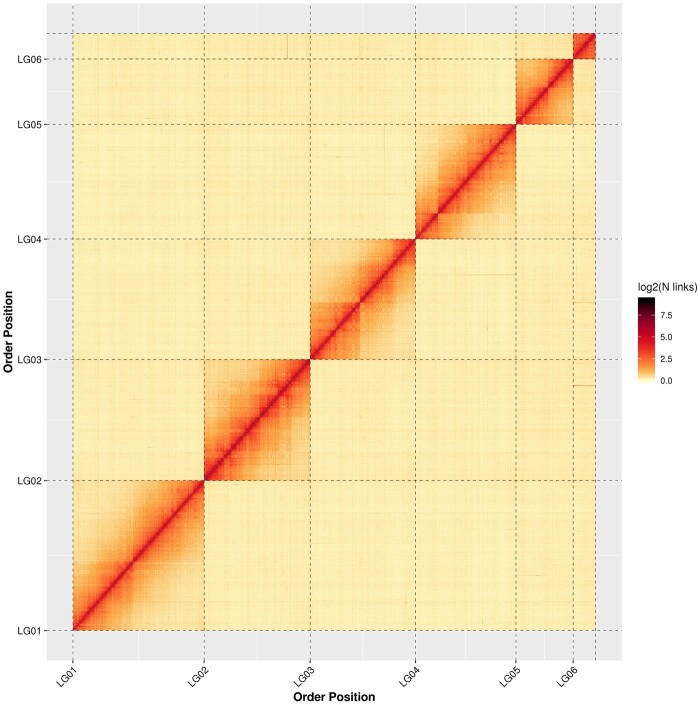
Contact matrix image of *Xya riparia* based on Hi-C data. The red color displays a high contact density and the yellow color displays a low contact density.

In all, 1.66 Gb of the genome sequences were anchored to six groups of chromosomes, accounting for 99.44% of the whole-genome assembly. Among the anchored sequences, 1.58 Gb of the sequences were properly ordered and oriented, accounting for 95.63% of the total chromosomal sequence length (supplementary table 1, Supplementary Material online). The result of the Hi-C assembly also showed that the corrected contig N50 was 4.18 Mb, the longest contig was 18.83 Mb, the scaffold N50 was 319.09 Mb, and the longest scaffold was 397.77 Mb (table 1 and supplementary table 2, Supplementary Material online). A genome scale heatmap was generated based on the assembly (fig. 1) in which the six chromosomes could be easily identified.

### Repeat Annotation, Gene Prediction, and Function Annotation

In all, 714.02 Mb of repetitive sequences from *X. riparia* were obtained based on the genome assembly. A total of 2,127,304 retroelements (Class I) and 961,469 DNA transposons (Class II) were identified. These two types of repetitive sequences accounted for 30.99% and 13.29% of the genome assembly, respectively. Of all the repetitive sequences, large retrotransposon derivatives and long terminal repeats in Class I accounted for 12.18% and 10.56% of the assembly, respectively, and terminal inverted repeats in Class II accounted for 11.44% (supplementary table 3, Supplementary Material online). The above three types of repetitive sequences were dominant among all the repetitive sequences. The results also suggested that *X. riparia’s* genome is highly repetitive and complex.

In total, 18,733 protein-coding genes were predicted. The average gene length, average exon length, average intron length, and average coding sequencing length were 18,646, 2,418, 16,227, and 1,582 bp, respectively (supplementary table 4, Supplementary Material online). For noncoding RNA sequences, 43 miRNA, 60,317 tRNA, 282 snRNA, and 205 rRNA were predicted, respectively (supplementary table 5, Supplementary Material online). By comparing the predicted genes with the Non-Redundant Protein Sequence Database (NR), Clusters of Orthologous Groups for Eukaryotic Complete Genomes (KOG), Gene Ontology (GO), Kyoto Encyclopedia of Genes and Genomes (KEGG), and TrEMBL, a total of 16,468 genes were successfully annotated to at least one of the databases above, and 87.91% of the *X. riparia* genome was finally functionally annotated (supplementary table 6, Supplementary Material online).

## Conclusion

In this study, the first reference genome of pygmy mole crickets was assembled at the chromosomal level. We found that the assembled genome size of *X. riparia* is 1.67 Gb. Furthermore, the study revealed that the contig N50 is 4.18 Mb, the longest contig is 18.8 Mb, the scaffold N50 is 319.1 Mb, and the longest scaffold is 397.8 Mb. These results indicate that both nanopore sequencing and Hi-C technology are effective tools for nonmodel genome assemblies. The genome data of *X. riparia* can serve as an important resource to facilitate further studies not only on pygmy mole crickets, but also on the entire orthopteran order.

## Materials and Methods

### Sampling and Sample Processing

The living female individuals of *X. riparia* were collected from Lyuxin Park in Leshan, Sichuan, China and were treated by starvation for 24 h to empty their digestive tracts. All specimens were kept alive and then transferred to the lab for further processing.

### Genome Size Estimation

The genome size of *X. riparia* was estimated via the *k*-mer approach (Liu et al. 2013). The genomic DNA was first extracted and sonicated into 350 bp fragments. After fragmentation, we then constructed the library followed by terminal repairs, the addition of poly As and adaptors, the selection of target fragments, and PCR (He et al. 2016). The constructed library was then qualified via Agilent 2100 and qPCR methods (Simbol et al. 2013). After qualification, the library was fixed on the microarray by conducting bridge PCR before sequencing on the Illumina NovaSeq 6000 platform (Lee 2021). Frequencies of 21-mers were generated based on 1.71 Gb of high-quality PE reads. The genome size was estimated using the following formula: *G* = *N_k_*_-mer_/*D*_average__*k*__-mer_, in which *G* represents genome size, *N_k_*_-mer_ represents total *k*-mer number, and *D*_average__*k*__-mer_ represents average *k*-mer depth (Guo et al. 2015).

### Nanopore Sequencing and De Novo Assembly

A total of 2 μg of genomic DNA was needed for nanopore sequencing (Lee et al. 2019). The genomic DNA was prepared using the NEB Next FFPE DNA Repair Mix kit (M6630, USA) and then processed with the ONT Template prep kit (SQK-LSK109, UK) following the manufacturer’s instructions (Kim et al. 2019). The library of large segments was premixed with loading beads and subsequently moved into a previously used and washed R9 flow cell using a pipette (Koivunen 2019). The library was sequenced on the ONT PromethION platform with the R9 cell and ONT sequencing reagent kit (EXP-FLP001.PRO.6, UK) following the manufacturer’s instructions.

Three different software programs were used for the de novo genome assembly: Canu (Koren et al. 2017) was used for the error correction of the clean data, Smartdenovo (Pu et al. 2020; Liu et al. 2021) was used for the genome assembly, Racon (Vaser et al. 2017) was used for the calibration referring to the nanopore sequencing data. After assembly, Pilon (Walker et al. 2014; Simão et al. 2015) was used for the calibration based on the Illumina sequencing data. The assembly assessment was performed through BUSCO (Simão et al. 2015).

### Chromosomal-Level Genome Assembly by Hi-C Data

Before the assembly, we performed an error correction. In brief, contigs were first broken into fragments of 50 kb and reassembled with reference to the Hi-C data. The regions that could not be restored to the original assembly were listed as candidate error regions. Positions of low Hi-C depth were considered as incorrect positions. After the initial correction, the corrected genome was assembled using LACHESIS software (Burton et al. 2013) with the following parameters:

CLUSTER_MIN_RE_SITES = 100;

CLUSTER_MAX_LINK_DENSITY = 2;

CLUSTER_NONINFORMATIVE_RATIO = 2;

ORDER_MIN_N_RES_IN_TRUN = 125;

ORDER_MIN_N_RES_IN_SHREDS = 124.

Finally, a heatmap of the entire genome was generated using the GGPLOT2 (Bian et al. 2020) package in R to assess the quality of the chromosomal-level genome assembly.

### Repeat Annotation, Gene Prediction, and Function Annotation

Through the application of LTR_FINDER (Xu and Wang 2007) and RepeatScout (Price et al. 2005) with default parameters, we constructed a repetitive sequence database of the genome on the basis of structural and ab initio predictions. We then applied the PASTEClassifier (Hoede et al. 2014) with default parameters to categorize the databases. The result was then merged with the database of Repbase (Jurka et al. 2005) and used as the eventual repetitive sequence database. Finally, we applied the RepeatMasker (Chen 2004) with the parameter -nolow -no_is -norna -engine wublast to predict the repetitive sequence of the genome based on the newly constructed reference genome.

The gene prediction of *X. riparia*’s genome was carried out by combining three approaches: ab initio prediction, homologous species prediction, and UniGene prediction. Ab initio predictions were performed using Genscan (Burge and Karlin 1997), Augustus v2.4 (Stanke and Waack 2003), GlimmerHMM v3.0.4 (Majoros et al. 2004), GeneID v1.4 (Alioto et al. 2018), and SNAP (Korf 2004) with default parameters. GeMoMa V1.3.1 (Keilwagen et al. 2016, 2018) with default parameters was used to predict homology-based species. The genome data of *Drosophila melanogaster, Zootermopsis nevadensis, Photinus pyralis*, and *Bicyclus anynana* were downloaded from Genbank for gene annotation. Hisat v2.0.4 (Kim et al. 2015) and Stringtie v1.2.3 (Pertea et al. 2015) with default parameters were used for assembly based on the reference transcriptome. TransDecoder v2.0 (Viricel et al. 2018) and GeneMarkS-T v5.1 (Tang et al. 2015) with default parameters were used for gene prediction. PASA v2.0.2 (Campbell et al. 2006) was used to predict UniGene sequences based on the nonreferenced assembly of transcriptome data and the parameter -align_tools gmap -maxIntronLen 20000. Finally, we used EVM V1.1.1 (Haas et al. 2008) with default parameters to integrate the results obtained by the three approaches mentioned above.

BlastN was used for the genome-wide alignment to identify microRNA and rRNA based on Rfam (Griffiths-Jones et al. 2005, http://rfam.xfam.org, last accessed January 2022). tRNAscan-SE (Lowe and Eddy 1997) with option -E –H was used to identify tRNA.

GO (Dimmer et al. 2012), KEGG (Kanehisaa and Goto 2000), KOG (Koonin et al. 2004), TrEMBL (Boeckmann et al. 2003), and NR (Marchler-Bauer et al. 2011) databases were used for alignment and to functionally annotate the predicted genes.

## Supplementary Material

Supplementary data are available at *Genome Biology and Evolution* online.

## Supplementary Material

evac001_Supplementary_Data
