# Correction to: A Chromosome-Level Genome Assembly of the Pygmy Mole Cricket *Xya riparia*

**DOI:** 10.1093/gbe/evac046

**Published:** 2022-04-13

Xiaolei Feng, Nan Yang, Qilu Wang, Hao Yuan, Xuejuan Li, Muhammad Majid, Xue Zhang, Chengquan Cao, Yuan Huang, A Chromosome-Level Genome Assembly of the Pygmy Mole Cricket *Xya riparia, Genome Biology and Evolution*, Volume 14, Issue 1, January 2022, evac001, https://doi.org/10.1093/gbe/evac001.

In the originally published version of this manuscript, the Data Availability section was incomplete. Another 3 files, including all data of genome assembly and annotations, are available on figshare, and can be accessed at https://doi.org/10.6084/m9.figshare.19336391.v1.

This error has been corrected.

