# Supplementary material for: A Chromosome-Level Genome Assembly of the Pygmy Mole Cricket Xya riparia
Source: Genome Biol Evol. 2022 Jan 6;14(1):evac001. doi: 10.1093/gbe/evac001 (PMC8765791; doi:10.1093/gbe/evac001)
Supplement: evac001_Supplementary_Data [file evac001_supplementary_data.docx]

Supplementary Materials for

A Chromosome-level Genome Assembly of the Pygmy Mole Cricket

*Xya riparia*

Xiaolei Feng, Nan Yang, Qilu Wang, Hao Yuan, Xuejuan Li, Xue Zhang, Chengquan Cao*, Yuan Huang*

**Supplementary Methods**

***DNA extraction, library construction, and Illumina sequencing***

The whole body of female *X. riparia* was used for DNA extraction. The sample was first stored in liquid nitrogen, and then it was moved onto dry ice. SDS-based lysis was applied for DNA extraction. The extracted DNA samples were purified by chloroform. The extracted genomic DNA was processed with ultrasonication and broken down into 350-bp fragments. After terminal repairing, adding poly-A and adaptors, target fragment selection, and PCR, the library was eventually constructed. The library used for further analysis was qualified by using Agilent 2100 and qPCR methods. The sequencing was performed using an Illumina NovaSeq 6000 sequencing platform.

***RNA extraction and transcriptome sequencing***

RNA was collected from mixed living samples of *X. riparia* by using TRNzol universal reagent. The library was constructed using the NEBNext® Ultra™ RNA Library Prep Kit (NEB, UK) according to the manufacturer’s instructions. Briefly, RNA of *X. riparia* was extracted using the Illustra RNAspin Mini RNA Isolation Kit (GE Healthcare, Hammersmith, UK) following the manufacturer's instructions. The isolated RNA was treated with RNase-free DNase I and eluted with RNase-free water, then fragmented following the first strand synthesis by adding the first strand synthesis reaction buffer and random primers. After finishing the second strand synthesis, end repair reaction buffer and end Rrep enzyme mix were used to repair the terminal and add poly-A. After adding the adaptors, target fragment selection and PCR were performed. A strand-specific library with an insert size of 250~350 bp was constructed, then sequenced using 150-bp paired-end reads using an Illumina NovaSeq 6000 sequencing platform.

***Hi-C analysis***

Hi-C assembly was performed based on the approach illustrated in Rao et al. and sequenced using an Illumina platform. The steps were as follows: Formaldehyde was used to fix the sample to cross-link intracellular proteins and DNA and to maintain the 3D structure inside the cell. The DNA was digested using the restriction endonuclease Hind III. Biotin-labeled bases were introduced to facilitate subsequent DNA purification and capture. Then, the interacted DNA was ligated. Finally, the DNA was purified and spit into 300–700 bp fragments. The fragmented DNA with interactive relationships was captured by streptavidin magnetic beads for library construction. High-throughput sequencing was performed using an Illumina platform with a read length of PE150.

**Supplementary Figures**


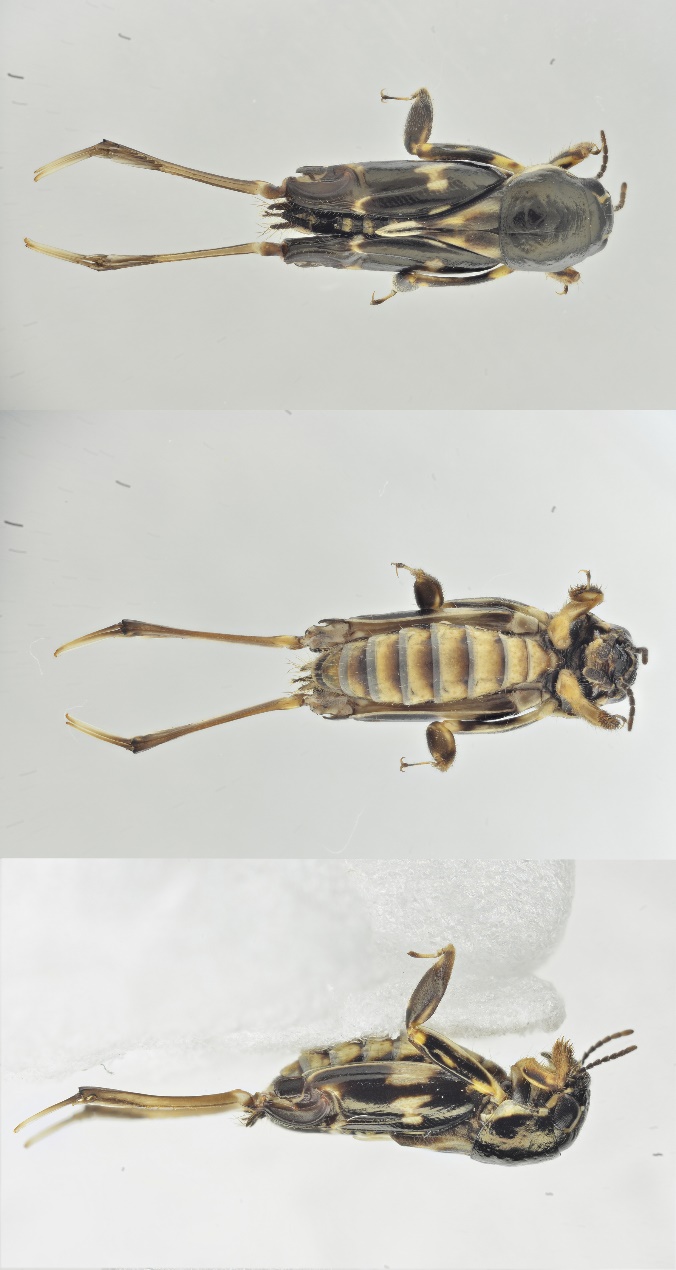


Figure S1. Pygmy mole cricket (*Xya riparia*)


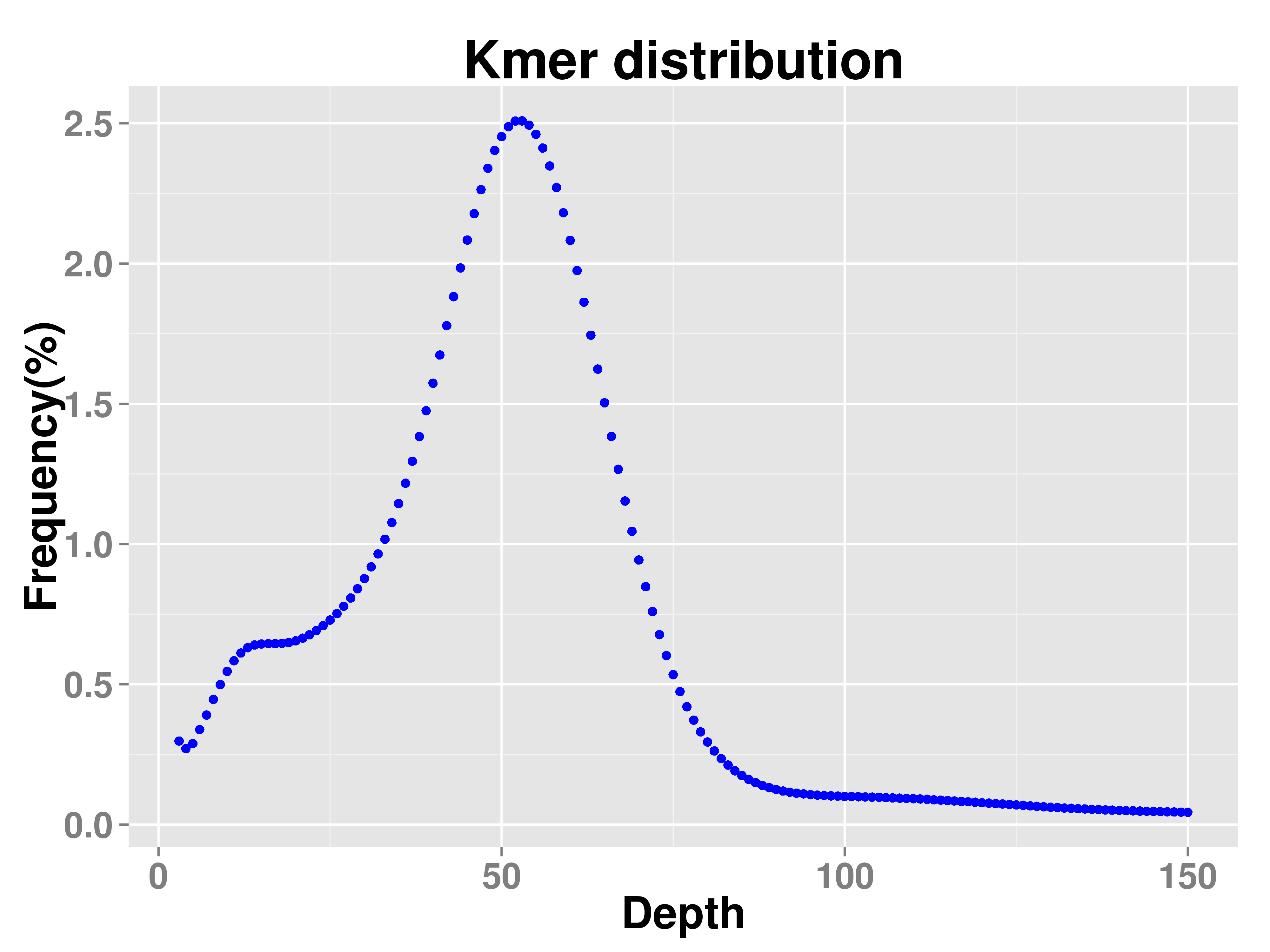


Figure S2. Frequency distribution of the 21-mer graph analysis used to estimate the genome size of *Xya riparia*

Table S1. Summary of Hi-C assembly data

| Group | Cluster Number | Cluster Length | Order Number | Order Length |
| --- | --- | --- | --- | --- |
| 01 | 283 | 419,437,598 | 155 | 397,759,120 |
| 02 | 155 | 330,670,179 | 99 | 321,022,224 |
| 03 | 188 | 329,697,765 | 114 | 319,079,044 |
| 04 | 237 | 323,869,632 | 121 | 304,422,261 |
| 05 | 94 | 179,288,023 | 52 | 173,100,756 |
| 06 | 55 | 72,886,409 | 28 | 68,153,308 |
| Total(Ratio%) | 1,012(98.25) | 1,655,849,606(99.44) | 569(56.23) | 1,583,536,713(95.63) |

Table S2. Summary of genome assembly and postassembly processing of *Xya riparia*

| Genome assembly of  *Xya riparia* | | Canu + Smartdenovo | Canu + Smartdenovo + Racon | Canu + Smartdenovo + Racon + Pilon | Canu + Smartdenov + Racon + Pilon + HiC |
| --- | --- | --- | --- | --- | --- |
| Summary of contigs | Contig Number | 947 | 947 | 947 | 1,030 |
|  | Contig Length | 1,623,774,976 | 1,664,671,396 | 1,665,235,322 | 1,665,235,322 |
|  | Contig N50 | 4,242,952 | 4,329,041 | 4,329,799 | 4,180,947 |
|  | Contig N90 | 1,041,256 | 1,079,359 | 1,080,240 | 988,259 |
|  | Maximum Length (bp) | 19,333,099 | 19,921,126 | 19,914,054 | 18,838,926 |
|  | GC Content (%) | 34.76 | 34.79 | 34.84 | 34.84 |
| Summary of scaffold | Total Number |  |  |  | 467 |
|  | Minimum Length |  |  |  | 1,000 |
|  | Scaffold Number |  |  |  | 467 |
|  | Scaffold Length |  |  |  | 1,665,291,622 |
|  | Scaffold N50 |  |  |  | 319,090,344 |
|  | Scaffold N90 |  |  |  | 173,105,856 |
|  | Maximum Scaffold |  |  |  | 397,774,520 |

Table S3. Summary of repetitive sequences of *Xya riparia* genome

| Type | Number | Length | Rate (%) |
| --- | --- | --- | --- |
| ClassI | 2,127,304 | 516,156,360 | 30.99 |
| ClassI/DIRS | 23,500 | 9,725,704 | 0.58 |
| ClassI/LARD | 1,048,489 | 202,862,916 | 12.18 |
| ClassI/LINE | 298,051 | 85,911,939 | 5.16 |
| ClassI/LTR/Copia | 31,794 | 14,562,247 | 0.87 |
| ClassI/LTR/Gypsy | 292,122 | 292,122 | 8.27 |
| ClassI/LTR/Unknown | 63,535 | 23,671,734 | 1.42 |
| ClassI/PLE | 360,266 | 96,673,614 | 5.81 |
| ClassI/SINE | 517 | 268,652 | 0.02 |
| ClassI/TRIM | 8,518 | 2,415,223 | 0.15 |
| ClassI/Unknown | 512 | 39,349 | 0.00 |
| ClassII | 961,469 | 221,328,620 | 13.29 |
| ClassII/Crypton | 2,507 | 182,284 | 0.01 |
| ClassII/Helitron | 52,989 | 14,064,290 | 0.84 |
| ClassII/MITE | 4 | 232 | 0.00 |
| ClassII/Maverick | 22,059 | 14,555,978 | 0.87 |
| ClassII/TIR | 831,494 | 190,453,146 | 11.44 |
| ClassII/Unknown | 52,416 | 5,891,306 | 0.35 |
| Potential Host Gene | 13,071 | 3,088,647 | 0.19 |
| SSR | 3,967 | 633,260 | 0.04 |
| Unknown | 393,291 | 72,628,182 | 4.36 |
| Total | 3,499,102 | 714,022,229 | 42.88 |

Table S4. Summary of gene information of *Xya riparia*

| Type | Number | Type | Number | Type | Number |
| --- | --- | --- | --- | --- | --- |
| Gene Number | 18,733 | Average Exon Length | 2,418.46 | Average CDS Number | 6.25 |
| Gene Length | 349,301,583 | Average Exon Number | 6.44 | Intron Number | 101,938 |
| Average Gene Length | 18,646.32 | CDS Number | 117,092 | Intron Length | 303,996,518 |
| Exon Number | 120,671 | CDS Length | 29,646,030 | Average Intron Length | 16,227.86 |
| Exon Length | 45,305,065 | Average CDS Length | 1,582.56 | Average Intron Number | 5.44 |

Table S5. Summary of noncoding RNA of *Xya riparia*

| Type | | Copy Number | Average Length (bp) | Total Length (bp) | Percentage (%) |
| --- | --- | --- | --- | --- | --- |
| miRNA |  | 43 | 77.3 | 3,324 | 0.00020 |
| tRNA |  | 60,317 | 73.7 | 4,449,748 | 0.26721 |
| snRNA | All | 282 | 152.8 | 43,088 | 0.00259 |
|  | Splicing | 238 | 164.2 | 34,787 | 0.00209 |
|  | CD-box | 42 | 191.7 | 8,050 | 0.00048 |
|  | scaRNA | 2 | 125.5 | 251 | 0.00001 |
| rRNA | All | 205 | 1,012.4 | 207,547 | 0.01246 |
|  | LSU rRNA eukarya | 79 | 1,572.0 | 124,186 | 0.00746 |
|  | SSU rRNA eukarya | 68 | 1,118.0 | 76,022 | 0.00457 |
|  | 5S rRNA | 41 | 117.9 | 4,832 | 0.00029 |
|  | 5.8S rRNA | 17 | 147.5 | 2,507 | 0.00015 |

Table S6. Summary of functional prediction of protein coding genes

| Annotation database | Annotated number | Percentage (%) |
| --- | --- | --- |
| GO | 7,151 | 38.17 |
| KEGG | 7,151 | 40.57 |
| KOG | 11,583 | 61.83 |
| TrEMBL | 16,401 | 87.55 |
| Nr | 16,385 | 87.47 |
| All | 16,468 | 87.91 |
